# Best practices in robotic magnetic navigation-guided catheter ablation of cardiac arrhythmias, a position paper of the Society for Cardiac Robotic Navigation

**DOI:** 10.3389/fcvm.2024.1431396

**Published:** 2024-09-27

**Authors:** Anna M. E. Noten, Tamas Szili-Torok, Sabine Ernst, David Burkhardt, Diogo Cavaco, Xu Chen, Jim W. Cheung, Christian de Chillou, Eugene Crystal, Daniel H. Cooper, Maurizio Gasparini, Tamas Geczy, Konrad Goehl, Burkhard Hügl, Qi Jin, Priit Kampus, Pedram Kazemian, Muchtiar Khan, Ole Kongstad, Jarkko Magga, Darren Peress, Pekka Raatikainen, Alexander Romanov, Ole Rossvoll, Gurjit Singh, Radu Vatasescu, Sip Wijchers, Kohei Yamashiro, Sing-Chien Yap, J. Peter Weiss

**Affiliations:** ^1^ Department of Clinical Electrophysiology, Thorax Center, Erasmus Medical Center, Rotterdam, Netherlands; ^2^ Department of Internal Medicine, Cardiology Center, University of Szeged, Szeged, Hungary; ^3^ Royal Brompton and Harefield NHS Foundation Trust, National Heart and Lung Institute, Imperial College London, London, United Kingdom; ^4^ Texas Cardiac Arrhythmia Institute, St. David’s Medical Center, Austin, TX, United States; ^5^ Heart Rhythm Center, Hospital da Luz, Lisbon, Portugal; ^6^ Department of Cardiology, Rigshospitalet, University of Copenhagen, Copenhagen, Denmark; ^7^ Division of Cardiology, Weill Cornell Medicine, NewYork Presbyterian Hospital, New York, NY, United States; ^8^ Department of Cardiology, CHU de Nancy, University Hospital Nancy, Nancy, France; ^9^ Sunnybrook Health Sciences Centre, University of Toronto, Toronto, ON, Canada; ^10^ Cardiovascular Division, Washington University School of Medicine, St. Louis, MO, United States; ^11^ Department of Cardiology, Humanitas University Hospital, Rozzano, Italy; ^12^ Department of Internal Medicine, Division of Cardiology, Medical University of Graz, Graz, Austria; ^13^ Department of Electrophysiology, Klinikum Nürnberg Süd, Nuremberg, Germany; ^14^ Department of Cardiology and Rhythmology, Marienhaus Klinikum St. Elisabeth, Neuwied, Germany; ^15^ Department of Cardiology, Shanghai Ruijin Hospital, Shanghai Jiao Tong University School of Medicine, Shanghai, China; ^16^ Department of Cardiology, North Estonian Medical Centre, Tallinn, Estonia; ^17^ Deborah Heart and Lung Center, Browns Mills, NJ, United States; ^18^ Department of Cardiology, Onze Lieve Vrouwe Gasthuis, Amsterdam, Netherlands; ^19^ Department of Cardiology, Lund University, Lund, Sweden; ^20^ Department of Cardiology, Oulu University Hospital, Oulu, Finland; ^21^ Pima Heart Physicians, PC, Tucson, AZ, United States; ^22^ Heart and Lung Center, Helsinki University Central Hospital, Helsinki, Finland; ^23^ E. Meshalkin National Medical Research Center of the Ministry of Health of the Russian Federation, Novosibirsk, Russia; ^24^ Department of Cardiology, St'Olavs University Hospital, Trondheim, Norway; ^25^ Division of Cardiology, Henry Ford Health System, Detroit, MI, United States; ^26^ Cardiology Department, Clinical Emergency Hospital, Bucharest, Romania; ^27^ Heart Rhythm Center, Takatsuki General Hospital, Osaka, Japan; ^28^ Department of Cardiology, Banner University Medical Center, The University of Arizona College of Medicine-Phoenix, Phoenix, AZ, United States

**Keywords:** robotic magnetic navigation, catheter ablation, atrial fibrillation, ventricular arrhythmia, remote magnetic navigation, ventricular tachycardia, premature ventricular beat, robotic navigation

## Abstract

**Preamble:**

Robotic magnetic navigation (RMN)-guided catheter ablation (CA) technology has been used for the treatment of cardiac arrhythmias for almost 20 years. Various studies reported that RMN allows for high catheter stability, improved lesion formation and a superior safety profile. So far, no guidelines or recommendations on RMN-guided CA have been published.

**Purpose:**

The aim of this consensus paper was to summarize knowledge and provide recommendations on management of arrhythmias using RMN-guided CA as treatment of atrial fibrillation (AF) and ventricular arrhythmias (VA).

**Methodology:**

An expert writing group, performed a detailed review of available literature, and drawing on their own experience, drafted and voted on recommendations and summarized current knowledge and practice in the field. Recommendations on RMN-guided CA are presented in a guideline format with three levels of recommendations to serve as a reference for best practices in RMN procedures. Each recommendation is accompanied by supportive text and references. The various sections cover the practical spectrum from system and patient set-up, EP laboratory staffing, combination of RMN with fluoroscopy and mapping systems, use of automation features and ablation settings and targets, for different cardiac arrhythmias.

**Conclusion:**

This manuscript, presenting the combined experience of expert robotic users and knowledge from the available literature, offers a unique resource for providers interested in the use of RMN in the treatment of cardiac arrhythmias.

## 1 Preamble

**Figure 1:**
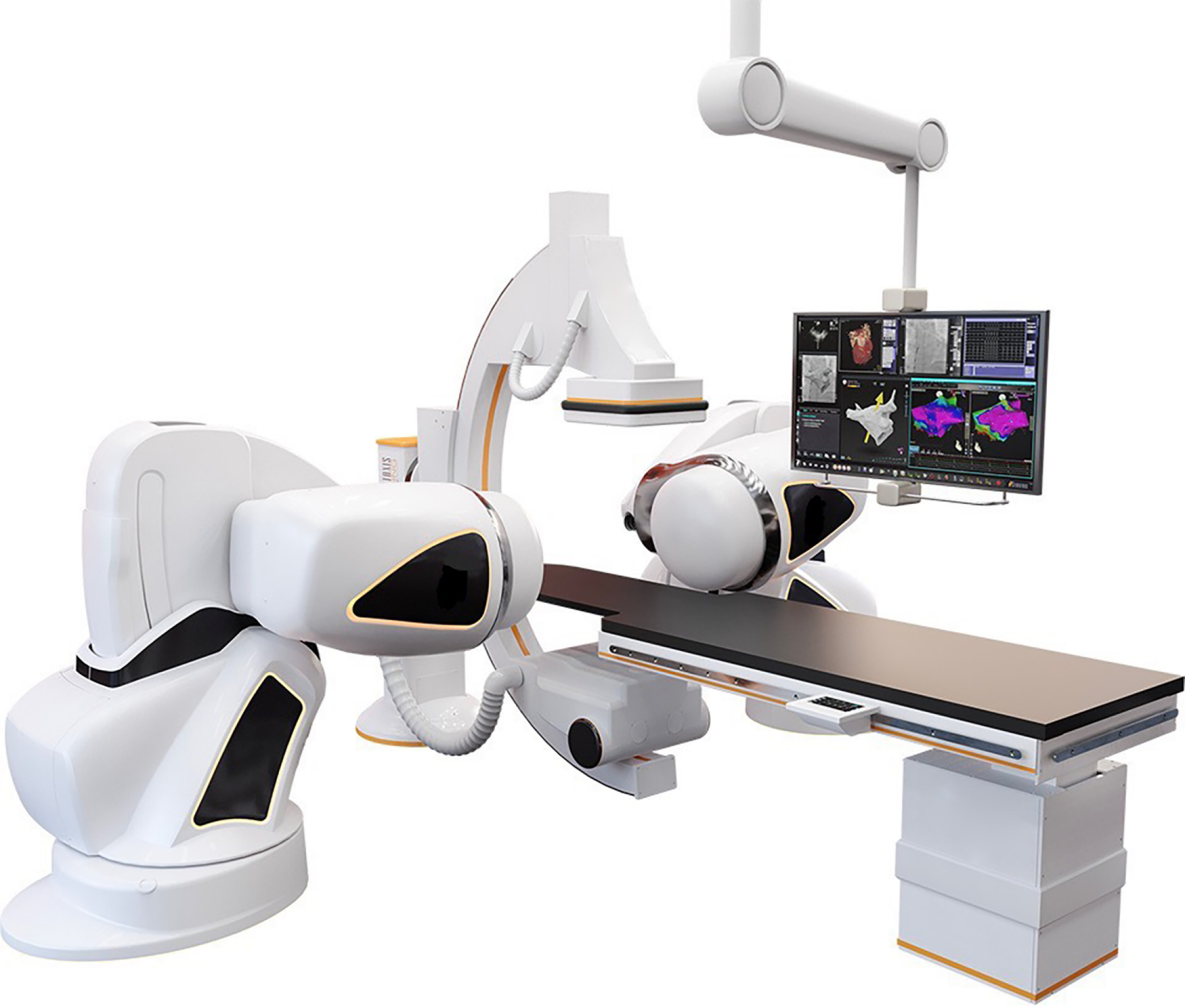
The RMN system. This figure illustrates the RMN Stereotaxis Genesis system hardware. The operation table and fluoroscopy system are present as they are in conventional invasive cardiology procedure labs. In addition, two external magnets are positioned alongside the patient which provide a magnetic field of 0.08–0.10 T. The magnetic vector is altered by the operator from the computer in the control room.

Robotic Magnetic Navigation (RMN)-guided catheter ablation (CA) has been used in the treatment of cardiac arrhythmias for almost 20 years (1, 2). Although initially various manufacturers have developed RMN technologies, Stereotaxis (Stereotaxis Inc., St. Louis MO, USA) provides the currently most frequently utilized system worldwide (1–3). In RMN, movement of the ablation catheter throughout the heart is guided robotically instead of manually, utilizing magnetic fields that interact with the magnetically enabled tip of the ablation catheter, causing the catheter to align parallel to the magnetic field (1). Figure 1 provides an illustration of a common RMN system.

The system has brought the ability of remote navigation and ablation which presents numerous advantages over manual ablation. The atraumatic, flexible designed catheter is drawn towards the myocardial surface with magnetic force and moves along with myocardial contractions, avoiding excessive distension of the cardiac wall that sometimes occurs unintentionally during manual manipulation of conventional catheters, which by construction must be relatively stiff and inflexible. Therefore, RMN allows for high catheter stability, improved lesion formation and a superior safety profile (4–6). Moreover, the RMN system presents the ability of uniform repetitive movements and the reach of anatomically difficult structures (7). The advantages of these characteristics have been demonstrated in clinical studies evaluating RMN performance, although there is lack of large randomized controlled trials comparing RMN with conventional ablation techniques.

So far, no guidelines or consensus papers on RMN-guided CA have been published. Therefore, we aimed to evaluate and describe common practice recommendations for CA procedures performed with this technology. The purpose of the present best practices statement is to consolidate the knowledge of expert users and provide recommendations on management of arrhythmias using RMN-guided CA.

## 2 Methodology

This consensus statement provides best practice recommendations for RMN-guided AF and VA ablation. An expert writing group, performed a detailed review of available literature, and drawing on their own experience, drafted recommendations and summarized current knowledge and practice in the field. Recommendations on RMN-guided CA are presented in a guidelines format with three levels of recommendations to serve as a reference for best practices in RMN procedures. Each recommendation is accompanied by supportive text and references. Recommendations are based on the available literature, as well as expert opinion. Expert opinion was evaluated systematically by an analysis of the consensus level between key-robotic users participating in the expert writing group.

The expert writing group consisted of RMN-utilizing electrophysiologists from high-volume centers, who were contacted by the independent Society of Cardiac Robotic Navigation (SCRN) and requested to participate in two extensive surveys on the use of RMN in AF ablation and VA ablation. All sites with an operational RMN system worldwide who fulfilled specific volume criteria were approached. Respondents were eligible to participate in the AF expert panel when they performed >50 procedures/year from 2015 onwards, whereas for VA this was defined as >30 procedures/year from 2015 onwards. Participants were eligible to participate in both panels as long as they fulfilled the volume criteria for each of the given arrhythmias.

### 2.1 Evidence

Consensus statements are evidence-based and derived primarily from published data. However, at present there are only a limited number of studies and no previous consensus statements describing best of practice for RMN-guided CA. A detailed review of literature was performed evaluating the available studies on RMN-guided CA. However, there are only few randomized studies on the technology. Recommendations are based on the available published data primarily. However, when there was lack of qualitative evidence, statements are “experience-based” and recommendations are therefore based on the level of consensus (LoC) between expert robotic users. The consensus level between respondents was determined using the following cut-offs: Good: ≥80% agreement, Moderate: 50%–80% agreement, Poor: <50% agreement amongst respondents. Recommendations for RMN-specific best practices are based on these three levels of consensus (LoC) between the participants and categorized as: “recommended”, “may be considered” and “not recommended”. Similarly to several electrophysiology related consensus documents [e.g., the 2017 SVT consensus document and the consensus statement on arrhythmias in congenital heart disease (8)], we have opted for an easy and user-friendly system of ranking using “colored hearts” that should allow physicians to easily assess current status of evidence/consensus (Table 1). Regarding general (non-RMN specific) electrophysiological practice, the considerations of the expert robotic users are of descriptive nature (i.e., “used by majority” or “used by minority”). They are presented to illustrate the current expert robotic practice with respect to several topics defined in the 2017 HRS/EHRA/ECAS/APHRS/SOLAECE expert consensus statement on catheter and surgical ablation of atrial fibrillation and the 2019 HRS/EHRA/APHRS/LAHRS expert consensus statement on catheter ablation of ventricular arrhythmias (9, 10).

**Table 1:** Scientific rationale of recommendations.

RMN specific recommendations			General (non-RMN specific) considerations		
Definition	Consensus statement	Symbol	Definition	Consensus statement	Symbol
Scientific evidence and/or consensus that recommendation is beneficial and effective. Requires at least 1 randomized trial, or strong observational evidence, or strong (≥80%) agreement between the expert panel members.	Recommended/indicated	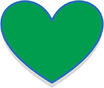	Consensus between authors that the consideration is beneficial and effective. The consideration is used by the majority of expert panel members (≥ 50%).	Used by a majority	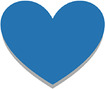
General agreement and/or scientific evidence that favors usefulness/efficacy of the recommendation. Supported by limited observational or randomized studies with smaller inclusion numbers and/or moderate agreement between expert panel members (50–80%).	May be used/may be recommended	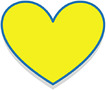	No general agreement between authors that consideration is beneficial and effective. The consideration is used by a minority of expert panel members (<50%).	Used by a minority	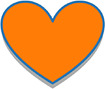
Scientific evidence that recommendation is not clearly beneficial and effective, or only used by a minority or great controversy between expert panel members (poor agreement <50%).	Used by a minority	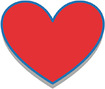			

## 3 The RMN expert panels

The AF expert panel consists of 14 respondents, who all performed RMN-guided AF ablation on a regular basis. Average experience with RMN-guided AF ablation was a median of 7.0 (IQR 4.0–10.0, range 2.0–11.0) years. All 14 respondents (100%) performed >50 AF ablation procedures in the previous 12 months with the RMN system. Ten respondents (71%) knew most of the features of the RMN system and occasionally relied on the manufacturer's help, whereas 4 (29%) knew all features independently.

The VA expert panel consists of 17 respondents who have performed RMN-guided VA ablation for a median of 8.0 (IQR 5.0–10.5, range 4.0–13.0) years. In the past 12 months, 5 (29%) operators performed >50 VA ablation procedures and 12 (71%) performed 30–50 procedures. Twelve respondents (71%) knew most of the RMN features and occasionally relied on the manufacturers help, whereas 5 (29%) independently operated all features.

General recommendations on RMN-guided CA presented in section 4, are evaluated by all individual AF and VA expert panels members. As four expert robotic users participated in both the AF and VA questionnaires, the total expert panel consisted of 27 individual respondents.

## 4 General recommendations on RMN-guided CA

### 4.1 EP laboratory staff

The HRS Heart Rhythm Society Expert Consensus Statement on Electrophysiology Laboratory Standards, presents recommendations on the EP laboratory staffing for ablation procedures and their training (11). These recommendations are also applicable to RMN-guided CA procedures. Additional specific RMN EP recommendations on laboratory staffing are presented in Table 2.

**Table 2:** General recommendations on RMN-guided catheter ablation.

Recommendation	Level of consensus	Symbol	References
Staff			
It is recommended to work with a dedicated clinical team to operate the RMN system and care for the patient	24/27 (89%)	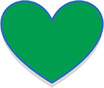	11
It is recommended that the clinical team is specifically trained to operate the RMN system	26/27 (96%)	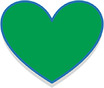	11
The clinical team may consist both off registered nurses, as well as registered technicians	16/27 (59%)	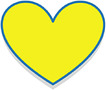	EO
Manufacturer's support may be considered to assist the staff to operate the RMN system.	18/27 (67%)	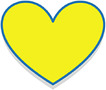	EO
Preparation and positioning of the patient			
It is recommended that the patient is set up on the table by either dedicated nurses and/or technicians	27/27 (100%)	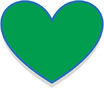	EO
Arm boards are recommended to support of the arms of the patient during the procedure	22/27 (81%)	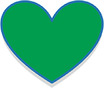	EO
Other types of arm support may be considered (e.g., soft Velcro wraps, fixation with blanket, angled arm support and gel pads)	14/27 (52%)	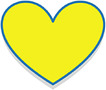	EO
Positioning of patients for left-sided ventricular targets is not more challenging than for other targets.	14/17 (82%)	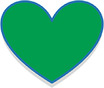	EO
It may be considered to iso-center the chamber of interest before catheters are inserted	17/27 (63%)	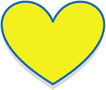	EO
It may be considered to perform pre-procedural imaging (either with CT or MRI) for anatomical and/or substrate representation, and image integration	21/27 (78%)	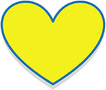	9, 10, 13, 14
It is recommended to use a mechanical ventilator that is MRI compatible, or otherwise place it outside the 5 Gauss zone	22/27 (81%), 5/5 (100%), respectively	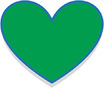	EO
Fluoroscopy			
The Siemens and Philips fluoroscopy systems are compatible with RMN	27/27 (100%)	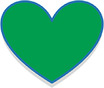	EO
It may be considered to take and store fluoroscopy images for use in Navigant software of the RMN system	17/27 (63%)	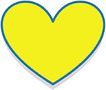	EO
It may be considered to use catheter overlay fluoroscopy images to reduce radiofrequency exposure	16/27 (59%)	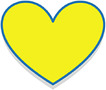	EO
Point annotation on fluoroscopy is used by a minority to visualize structures or equipment	13/27 (48%)	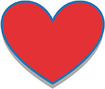	EO

EO, expert opinion; MRI, magnetic resonance imaging; RMN, robotic magnetic navigation.

At the majority of centers performing RMN-guided CA [24/27 (89%)], a dedicated clinical EP laboratory team works at the RMN laboratory, which is recommended. In some centers the technicians are dedicated but nurses are not [1 (4%)], or the staff is dedicated but on rotation with other cardiovascular departments [2 (7%)]. The median tenure of the staff of the expert panel is 5.0 (IQR 3.5–10.0, range 2.0–20.0) years. It is recommended that the laboratory staff is specifically trained to employ the RMN system [LoC 26/27 (96%)]. It may be considered to compose laboratory staff teams of both registered nurses as well as registered technicians [LoC 16/27 (59%)]. A minority of lab teams [9 (33%)] operate all equipment independent of manufacturer support. Eighteen teams (67%) occasionally receive assistance from the manufacturers to operate the equipment.

### 4.2 Preparation and positioning of the patient

Pre-operative examinations, including assessment of the medical history, physical examination, ECG recordings, relevant imaging and laboratory tests and medication use, should be performed as in any invasive EP procedure and recommendations are described in detail elsewhere (11). In most patients, anti-arrhythmic drugs are stopped 5 half-lives before the procedure to allow the target arrhythmia to be induced (11). Discontinuation of anticoagulant therapy should be contemplated based on the thromboembolic and bleeding risk (9, 11). Nowadays, AF ablation procedures are frequently performed with uninterrupted anticoagulant therapy, and treatment with DOAC results in less bleeding complications, compared to uninterrupted warfarin (9, 12). This also applies to RMN guided ablation.

**Figure 2:**
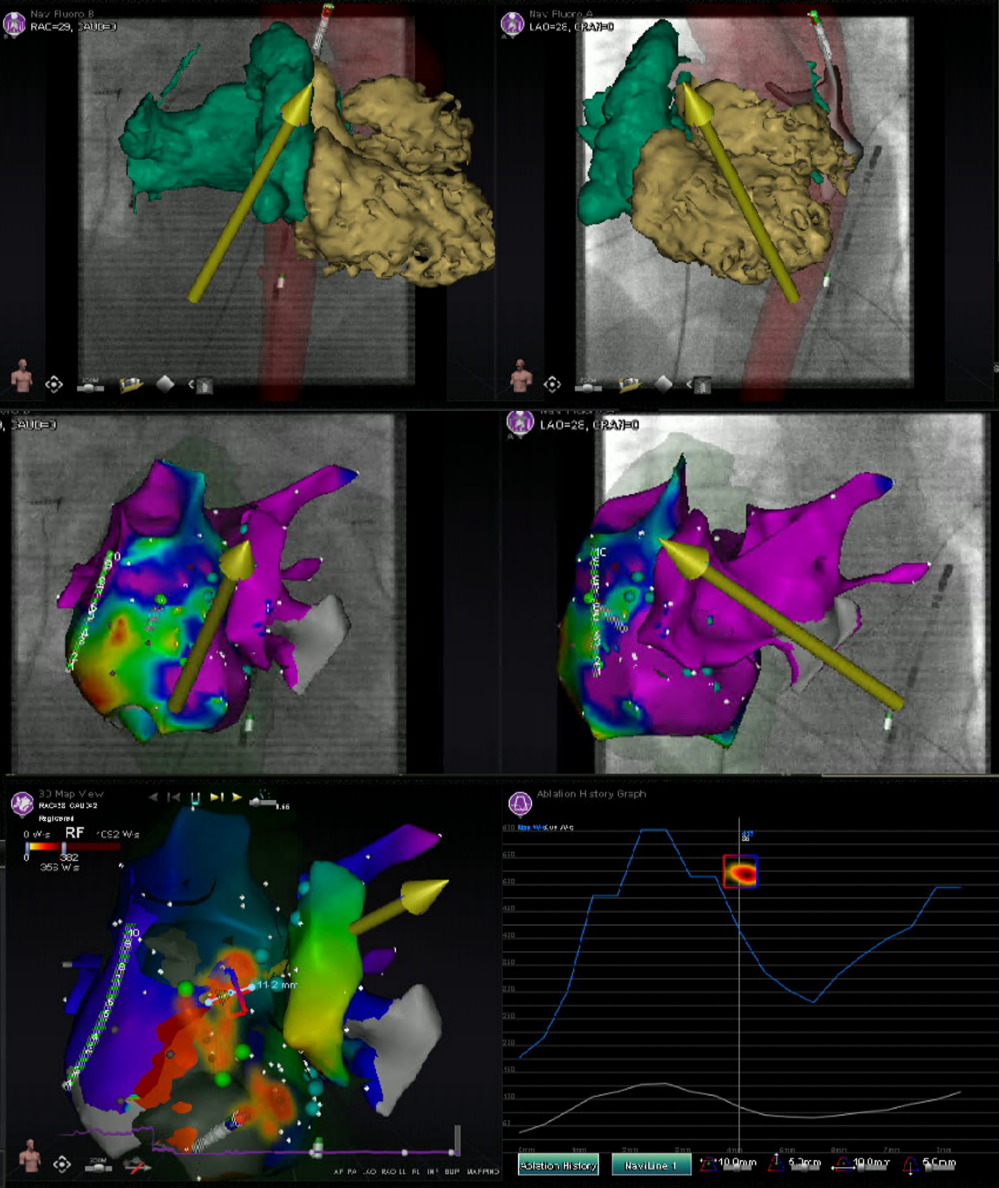
Image integration. This figure shows the various screen outputs which are displayed in real-time to the operator during a RMN-guided ablation procedure utilizing image integration in a patient after congenital heart surgery. This patient had a single ventricle physiology, with a small RV, large ventricular septal defect and double inlet left ventricle with two AV valves. The patient previously underwent total cavo-pulmonary connection (TCPC) and the remaining RA is rather small. The upper panels show segmented 3D CMR scan images overlayed on the fluoroscopy images. The RMN magnetic vector is displayed as yellow arrow and the ablation catheter is also visualized. The middle panels show the CARTO bipolar voltage maps in two directions of the ventricle together with the RMN magnetic vector (yellow arrow) and the ablation catheter (retrograde approach). The native chambers have normal voltage, whereas the TCPC is large and has scar and that is where reentry was observed and atrial tachycardia originated. The left lower panel visualizes the applied therapy using the Ablation History feature's output on the activation map. The applied therapy is displayed in yellow-orange (see Figure 3 for a more detailed explanation). The right lower panel provides the so called Ablation History graph visualizing the applied energy. The various imaging modalities displayed are all fully integrated with the RMN system.

Pre-procedural evaluation of presence of intracardiac thrombus by TTE and/or TOE, is advised according to the 2017 and 2019 HRS/EHRA/ECAS/APHRS/SOLAECE expert consensus statements (9, 10). Pre-procedural imaging with cardiac CT, nuclear imaging and/or CMR can be of value for anatomical and/or substrate visualization [e.g., the number of pulmonary veins (PVs), anatomical variants of PVs and the arrhythmogenic substrate by visualization of the location and extent of late enhancement] (9, 10). Regarding RMN-guided procedures, the majority of respondents [21 (78%)] perform pre-procedural imaging (either with CT or MRI) for visualization of the anatomy and/or substrate identification. Furthermore, cardiac CT, nuclear imaging as well as CMR allow for image integration with electro-anatomic mapping (EAM) (13, 14). Integration of ventricular myocardial scar imaging with EAM has further contributed to the ability to recognize and eliminate disrupted and potentially slowly conducting regions of myocardium that for instance are critical to the maintenance of VT (13). In RMN-guided CA VA ablation image integration is increasingly more frequently used to identify and target complex substrate in real-time (Figure 2) (10, 15). Image integration allows electrophysiologists to target more complex arrhythmias and also significantly improves the efficacy, efficiency, and safety profile of these procedures (16).

RMN specific recommendations on the positioning of the patient are presented in Table 2. It is recommended by the majority of respondents [LoC 27/27 (100%)] that in RMN-guided CA the patient is positioned on the table by either dedicated nurses [20 (74%)] and/or technicians [7 (26%)], e.g., to prevent mispositioning of mapping system's patches and locator pads and to avoid map shifts during the procedure. In general, arm boards are recommended to support the patient's arms during the procedure without interference with the movement of the magnets [LoC 22/27 (81%)]. Other types of arm support may be considered [LoC 14/27 (52%)]. Suggested other types of arm support include: soft Velcro wraps (Velcro Inc., Manchester, NH, USA) [7 (26%)], fixation with blanket [2 (7%)], angled arm support [1 (4%)], gel pads [1 (4%)], the AliMed Radiolucent Abdominal Immobilizer (Alimed Inc., Dedham, MA, USA) [1 (4%)] or specially designed plexi support [1 (4%)]. It may be considered to isocenter the cardiac chamber of interest before the catheters are inserted [LoC 17/27 (63%)]. There is general agreement [LoC 14/17 (82%)] that it is not more challenging to prepare the patient for LV procedures compared to other cardiac targets.

In RMN-guided procedures performed under general anesthesia, it is recommended to use a mechanical ventilator which is MRI compatible [LoC 22/27 (81%)]. If no MRI compatible mechanical ventilator is available, it is recommended to be placed outside the 5 Gauss zone [LoC 5/5 (100%)]. Invasive blood pressure monitoring is not standard of practice of respondents in AF ablation, but used occasionally for instance in patients with tenuous hemodynamics. Regarding VT ablation, invasive blood pressure monitoring is generally applied in patients with LV dysfunction. Placement of urinary tract (Foley) catheter is considered during expected lengthy procedures on a case by case basis.

### 4.3 Fluoroscopy

In interventional fluoroscopic imaging, the ALARA (as low as reasonably achievable) concept is widely adopted, striving to keep radiation dosages as low as possible (17). Data from multiple meta-analysis evaluating fluoroscopy data uniformly showed a significant reduction of fluoroscopy exposure in favor of RMN when compared to conventional techniques, which can be considered a major advantage of this CA technique (5, 6, 18–22).

Recommendations on the combination of fluoroscopy systems with RMN are presented in Table 2. Both the Siemens and the Philips fluoroscopy systems are compatible with the RMN technology. Nineteen respondents (70%) use a Siemens (Siemens Medical Solutions Inc., Malvern, PA, USA) and 8 (30%) use a Philips (Philips N.V, Eindhoven, The Netherlands) fluoroscopy system. Fluoroscopy imaging can be incorporated into several RMN features. It may be considered to take and store fluoroscopy images for use in the Navigant software (Stereotaxis Inc.) [LoC 17/27 (63%)]. It may be considered to use catheter overlay fluoroscopy images to reduce the radiofrequency exposure [LoC 16/27 (59%)]. However, point annotation on fluoroscopy is only used by a minority to visualize structures or equipment [LoC 13/27 (48%)].

### 4.4 Cost effectiveness

Installation of the RMN technology requires additional costs, e.g., to install the essential equipment, to train EP lab staff and for maintenance. The technology therefore could have variable accessibility in different healthcare systems worldwide. There is one study evaluating clinical and direct cost perspectives of RMN-guided ablation of adult AVNRT, using either RMN, conventional manual RF and cryoablation techniques (23). In this study, RMN and conventional manual RF appeared to be equally effective and associated with lower AVNRT recurrence rates when compared to cryoablation. This study observed significant disposable cost savings of conventional MAN when compared to RMN, despite similar efficacy (23). However, for other indications - such as VA ablation (See Section 6), the ablation of pediatric patients (24, 25) and those with congenital heart defects (26, 27) - there is evidence of improved performance of the RMN system, but there is no data of cost-effectiveness in other populations available yet. Besides, RMN reduces radiation exposure to the operator and eases operator fatigue. Whether these matters, justify the additional costs of RMN over conventional CA techniques remains a topic of ongoing debate.

## 5 Recommendations on RMN-guided atrial fibrillation ablation

### 5.1 General

Nowadays, RMN is increasingly used in the treatment of AF worldwide. The initial experience was reported in 2006 (28). Since then, a growing body of literature has been published evaluating RMN-guided AF ablation. However, there are no randomized trials comparing RMN with conventional techniques. Most of the non-randomized observational studies have been evaluated by multiple meta-analysis, covering different periods in time (5, 19–21, 29). Results from these meta-analysis are remarkably consistent showing comparable acute and long-term success rates between RMN and manual ablation. Furthermore, procedure and ablation times were significantly longer in RMN, whereas fluoroscopy exposure was reduced. Finally, major complication rates were significantly lower in RMN-guided ablation, especially with respect to pericardial effusion and tamponade (5, 19–21, 29).

Recommendations on RMN-guided AF ablation and general AF ablation considerations are presented in Tables 3, 4 respectively. The RMN system is considered feasible for the complete spectrum of AF ablation, including primary pulmonary vein isolation (PVI), redo PVI and the ablation of complex substrates [LoC 13/14 (93%)]. One respondent (7%) uses the RMN system only for redo procedures and complex anatomies.

**Table 3:** RMN specific recommendations on atrial fibrillation ablation.

Recommendation	Level of consensus	Symbol	References
General			
The RMN system is appropriate for standard first and redo AF ablation	13/14 (93%)	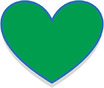	5, 19–21, 23, 24
The RMN system is appropriate for complex AF ablation, including complex substrate ablation	13/14 (93%)	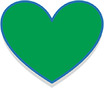	5, 19–21, 23, 24
Approach and Transseptal puncture			
It is recommended that vascular access is attained by the operating electrophysiologist or fellow only.	13/14 (93%)	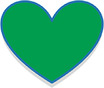	11
A transseptal approach is recommended to reach left-atrial targets	14/14 (100%)	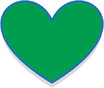	25, 26
A retrograde transaortic approach is rarely used in standard RMN-guided AF ablation	0/14 (0%)	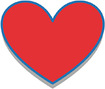	EO
Mapping			
The CARTO 3D mapping system is considered the first-choice mapping system for RMN-guided AF ablation	14/14 (100%)	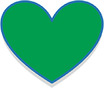	14
Automation features			
Various automation features are used by a minority in standard RMN-guided AF ablation. These include: the Automap, Bullseye, Vector lock, Naviline and Click and Go features	1/14 (7%)	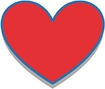	EO
The Vdrive is used by a minority for RMN-guided AF ablation	1/14 (7%)	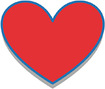	EO
RMN settings			
It may be considered to use a magnetic field strength of 0.1 T	10/14 (71%)	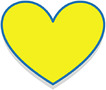	EO
It is only rarely needed to tilt the magnets for larger patients or steeper imaging angles	1/14 (7%)	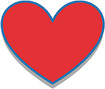	EO
It is recommended to use e-Contact Module during AF ablation, to evaluate whether the ablation catheter is in (optimal) contact with the myocardial wall tissue or not	9/10 (90%)	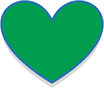	32, 33
It is recommended to use the Ablation History feature for real-time evaluation of lesion formation during RMN-guided AF ablation	13/14 (93%)	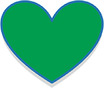	EO
Sheaths, catheters and manipulation			
It is recommended to use one of the following sheaths to guide the RMN catheter into the LA: SL0, SL1 or steerable sheath	12/14 (86%)	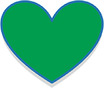	EO
It may be considered to use the introducer (i.e., haemo-adapter) to insert the RMN catheter into the sheath	9/14 (64%)	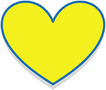	EO
It may be considered to manipulate the sheath during a RMN-guided AF ablation procedure to support catheter movement	9/14 (64%)	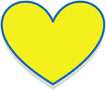	EO
To reach left-sided PV's, a sheath position close to the intra-atrial septum (<2 cm) may be considered	8/14 (57%)	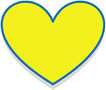	EO
It may be considered to “loop” or ‘pin and roll’ the catheter to approach the right-sided PV's	9/14 (64%)	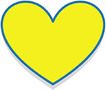	EO
To reach right-sided PV's, the sheath may be rotated, advanced or pulled back	7/14 (50%)	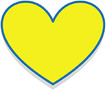	EO
It may be considered to use a sheath into the LA to guide manipulation of the diagnostic catheter	9/14 (64%)	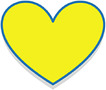	EO
The SmartAblate Stockert ablation generators are recommended for RMN-guided AF ablation	14/14 (100%)	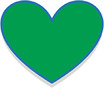	11
The Navistar Thermocool RMT catheter is recommended for RMN-guided AF ablation	12/14 (86%)	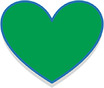	EO
It may be considered to navigate towards anatomical areas that are difficult to reach, prior to starting the ablation	11/14 (79%)	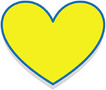	EO
Advancing and retraction of the RMN catheter is recommended by rolling the mouse wheel, although a minority uses the key pad or joy stick	13/14 (93%)	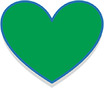	EO

AF, atrial fibrillation; EO, expert opinion; LA, left atrium; PV, pulmonary vein; RMN, robotic magnetic navigation.

**Table 4:** General considerations on atrial fibrillation ablation.

Recommendation	Level of consensus	Symbol	References
General			
Conscious sedation is more frequently used compared to general anesthesia during RMN-guided AF ablation	9/14 (64%)	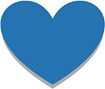	9
Approach and TSP			
In most cases, TSP is performed either at the central part of the foramen ovale or a more inferior-anterior position	11/14 (79%)	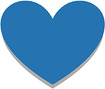	EO
Double transseptal access is achieved by double puncture, or recrossing	7/14 (50%), 7/14 (50%) resp.	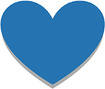	27
The majority of respondents place the diagnostic catheter in the top transseptal sheath	7/7 (100%)	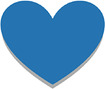	EO
Identification of the esophagus (e.g., by storing x-ray images of TOE probe) is done by a minority	4/14 (29%)	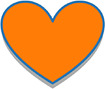	EO
Mapping			
Multi-electrode mapping catheters are used by the majority	10/14 (70%)	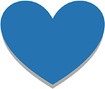	EO
Respiratory compensation is frequently used during mapping	12/14 (86%)	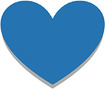	EO
The ‘adjust FAM with point-by-point mapping’ setting is used by a minority	4/14 (29%)	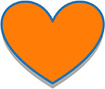	EO
The CARTO sound map feature is used by a minority	1/14 (7%)	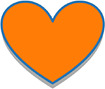	EO

AF, atrial fibrillation; CT, computed tomography; FAM, fast anatomical mapping; ICE, intracardiac echocardiography; MRI, magnetic resonance imaging; PV, pulmonary vein; PVI, pulmonary vein isolation; RMN, robotic magnetic navigation; TOE, transesophageal echocardiography; TSP, transseptal puncture.

According to present consensus statement, the type of anesthesia used for AF CA depends in part on the availability of anesthesia support. Given the need to minimize patient movement to improve mapping and catheter stability, deep sedation or general anesthesia are generally preferred during AF ablation (9). The majority of respondents [9 (64%)] perform RMN-guided AF ablation procedures under conscious sedation in preference to general anesthesia [which is used by five respondents (36%) as standard of care]. Half of the respondents (50%) have an anesthesiologist present during all AF ablation procedures. At other centers [7 (50%)], sedation may be performed by specifically trained nurses.

### 5.2 Approach and transseptal puncture

Anatomical considerations, equipment, techniques and challenges of gaining LA access by TSP have been well described (30, 31). TSP continues to be a challenging procedural step with inherent risks, mostly with respect to cardiac tamponade (31). Regarding RMN-guided AF ablation, all respondents perform TSP to reach the LA (LoC 14/14 (100%). In general, a retrograde transaortic approach is not a first-choice approach in RMN-guided AF ablation [LoC 0/0 (0%)]. TSP is performed at the central part of the foramen ovale or a more inferior-anterior position by the majority of respondents [11 (79%)]. Single TSP with passive recrossing was compared to double TSP in AF ablation, and there was no clear benefit of one technique over the other (32). Double TSP [7/14 (50%)] and recrossing [7/14 (50%)] techniques for the second transseptal access are equally used by the RMN AF expert panel. In case of double TSP, the diagnostic catheter is placed in the top sheath and the ablation catheter in the lower one [LoC 7/7 (100%)].

TSP can be performed either guided by fluoroscopy alone and/or guided by echocardiography imaging (intracardiac echocardiography (ICE) or transesophageal echography (TOE)) (9, 31). ICE guiding nowadays presents the ability of zero-fluoroscopy TSP (33). However, despite the potential value of ICE, it is important to recognize that clinical trials did not conclude that the use of ICE improves the safety of ablation procedures (31), whereas ICE substantially increases procedure costs, requires an additional vascular access, and additional training to manage (11). Regarding RMN-guided AF ablation, all respondents [14 (100%)] perform TSP either with TOE or ICE guidance. The majority of respondents [8 (57%)] perform TSP with ICE, and six (43%) preferably use TOE for guidance. There is controversy amongst the expert panel about the necessity of esophagus identification. Eight respondents (57%) do not advocate identification of the esophagus, but a minority of respondents store x-ray images of the TOE probe [4 (29%)].

### 5.3 Mapping

The RMN system has full mapping integration with two of the currently available 3D electroanatomic mapping systems: The CARTO 3D mapping system (Biosense Webster Inc., Diamond Bar, CA, USA) and the AcQMap (Acutus Medical Inc., Carlsbad, CA, USA) mapping system (Figure 2) (14). Other mapping modalities, such as the EnSite NavX (Abbott, Green Oaks, Il, USA) system, can also be combined with RMN, however only in parallel (i.e., not fully integrated) fashion. The CARTO 3D mapping system is considered the first-choice mapping system for RMN-guided AF ablation [LoC 14/14 (100%)]. The EnSite NavX and the AcQMap mapping systems are occasionally used by respondents. The novel version of the EnSite X EP mapping system, holds future possibilities to full integration.

During mapping, a median FAM resolution of 15.0 (IQR 12.0–17.0, range 11.5–20.0) is advised by the respondents. Three respondents comment that a resolution of 10.0–13.0 is sufficient to map the body of the LA and higher resolutions around 15.0–18.0 should be used for more detailed maps of insertions of the PVs and left atrial appendage (LAA). Multi-electrode mapping catheters (such as Lasso or Pentaray catheters) are frequently used [10 (71%)]. Respondents use the ablation catheter to refine the PV anatomy on the map, which can be of benefit when no pre-procedural imaging is present or if the PVs are difficult to canulate with multi-electrode mapping catheters. Respiratory compensation is frequently applied during mapping [12 (86%)]. The “adjust FAM with point-by-point mapping” setting is not common practice [4 (29%)]. The CARTO sound map is generally not used in RMN-guided AF ablation [1 (7%)].

### 5.4 Automation features and Vdrive

There are various automation features which can be used during RMN-guided CA. In general, these features are not often used for AF ablation. The following automation features are only infrequently used by respondents and not recommended for standard AF ablation: the Automap feature [LoC 2/14 (14%)], The Bullseye feature [LoC 8/14 (57%)], the Vector lock feature [LoC 6/14 (43%)], The Naviline feature [LoC 3/14 (21%)], the Click and Go feature [LoC 4/14 (29%)].

The Vdrive robotic catheter manipulation system (Stereotaxis, Inc.) consists of an electromechanical driving mechanism that can be adjunctively operated with magnetically driven catheters. It is designed to allow an operator to remotely advance, retract, rotate, and deflect a multipolar catheter. This has the potential to enable more fully remote procedures, reduce procedure time, and further reduce the operator's fluoroscopy exposure (34). Although its utility has been described, the Vdrive is used by a minority regarding standard RMN-guided AF ablation. In general, respondents think there is no harm from using this feature [LoC 14/14 (100%)]. However, only one respondent (7%) frequently uses it, five (36%) occasionally use it and 8 respondents (57%) do not have it installed, because of additional costs, complexity and questionable benefit.

### 5.5 RMN settings

It may be considered to preferentially use a magnetic field strength of 0.1 T instead of 0.08 T [LoC 10/14 (71%)]. However, the setting of 0.08 T is applicable to any patient (especially patients with increased axial chest dimensions and/or obesity because of more distance between the magnets). It is rarely needed to tilt the magnets for larger patients or steeper imaging angles [LoC 1/14 (7%)]. A median CAS step size of 3.0 mm (IQR 3.0–4.5, range 1.5–6.0) is recommended.

The e-Contact Module is a feature designed for RMN that measures the quality of catheter–tissue contact (35). The e-Contact Module provides real-time feedback whether the ablation catheter is in (optimal) contact with the myocardial wall tissue or not, incorporating electrical impedance variables, data on the cardiac induced motion of the catheter tip and on the torque being applied by the magnetic field (35). The e-Contact Module does not inform on the Contact Force applied by the magnetic guided catheter, as magnetic fields of 0.08 and 0.10 T provide quite stable catheter contact forces and the more flexible shaft of the RMN catheter by design buckles when higher forces are being applied (36). Contact feedback by the e-Contact Module further decreases fluoroscopy exposure and improves VT-free survival in RMN-guided ischemic VT ablation (37). Therefore, it is recommended to use the e-Contact Module during RMN-guided AF ablation [LoC 9/10 (90%)]. Four respondents (29%) do not have this relatively novel feature installed yet, which is not approved by the F.D.A. for use in the U.S.A. at the time of writing.

**Figure 3:**
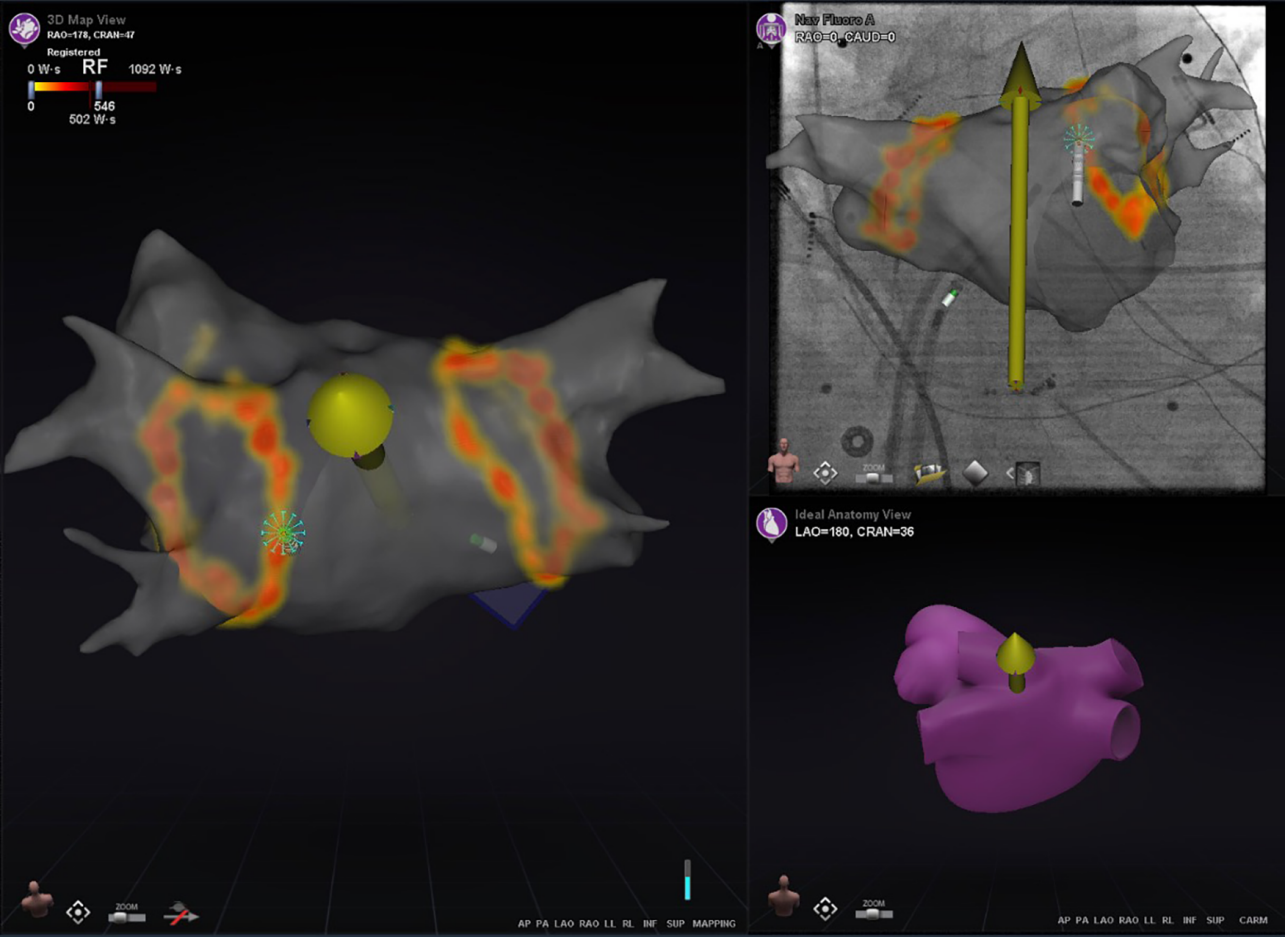
The ablation history feature. This figure illustrates the Ablation History of a patient who underwent pulmonary vein isolation. The pulmonary veins were isolated by applying two Wide Area Circumferential Ablation (WACA) lines around the left-sided and right-sided pulmonary veins. The main panel represent the CARTO screen, on which the LA FAM map is displayed and made transparent. The applied energy during ablation is visualized 3-dimensionally from yellow to orange based on the applied Watt-Seconds per location. In this case, the ablation catheter is in optimal contact with myocardial tissue, which is evaluated by the e-Contact Module and displayed real-time to the user with a dense blue starburst at the catheter tip.

Ablation History (Figure 3) provides a 3-dimensional visual display of the history of the catheter's power output and duration of energy application at each ablation location in the CARTO map. It uses incrementally darker shades of color ranging from light yellow to deep red corresponding to the increasing Watt*Seconds of energy delivered (Figure 3). It is recommended to use the Ablation History feature during RMN-guided AF ablation by almost all respondents [LoC 13/14 (93%)], which is used in real-time to evaluate the completeness of ablation lines in PVI. In redo AF procedures, the Ablation History of the preceding ablation is generally not used by respondents to strategize the second procedure [LoC 3/14 (21%)].

### 5.6 Sheaths, catheters and manipulation

It is recommended to use one of the following sheaths to guide the RMN catheter in the LA: SL0, SL1 (Abbott) or a steerable sheath [LoC 12/14 (86%)]. It may be considered to use the introducer (i.e., haemo-adapter) to insert the RMN catheter into the sheath [LoC 9/14 (64%)]. In addition, nine respondents (64%) consider manipulation of the sheath during a RMN-guided AF ablation procedure to support catheter movement. There is no consensus to what extent the sheath with the ablation catheter should be advanced into the LA. A sheath position close to the septum (<2 cm) may be considered to support the RMN catheter in targeting left-sided PVs [LoC 8/14 (57%)]. It may be considered to “loop” or “pin and roll” the catheter to approach the right-sided PVs [LoC 9/14 (64%)]. A combination of the following manipulations might also be considered to approach the right-sided PVs: rotate the sheath [LoC 8/14 (57%)], or advance the sheath [LoC 7/14 (50%)] or pull back [LoC 7/14 (50%)].

Regarding the diagnostic catheter, it may also be considered to use the sheath to guide diagnostic catheter manipulation in the left atrium [LoC 9/14 (64%)]. There is no consensus to what extent the diagnostic catheter's sheath should be advanced into the LA [LoC 1/14 (7%)].

General recommendations on the generator used in RF CA are presented elsewhere (11). It is recommended by the expert panel to use one of the various generations of the SmartAblate Stockert ablation generators (Johnson & Johnson/Biosense Webster) [LoC 14/14 (100%)]. The Navistar Thermocool RMT catheter (Biosense Webster) is most frequently used for RMN-guided AF ablation [LoC 12/14 (85%)]. Alternative ablation catheters suggested by respondents are: the Celsius RMT catheter (Biosense Webster) [LoC 1/14 (7%)] and the Navistar RMT 4 mm catheter (Biosense Webster) [LoC 1/14 (7%)].

It may be considered to navigate towards difficult anatomical areas prior to starting the ablation [LoC 11/14 (79%)]. Advancement and retraction of the RMN catheter is recommended by rolling the mouse wheel [LoC 13/14 (93%)]. A minority uses the key pad [LoC 2/14 (14%)] or joystick [LoC 3/14 (21%)].

The approach of right (inferior) pulmonary vein(s) is considered more difficult by some members of the AF expert panel. Expert panel members propose that “looping” of the catheter provides a solution for this [5 (36%)]. Otherwise advancing or retracting of the sheath [4 (29%)] and the use of a steerable sheath [3 (21%)] can be considered. Respondents mention that the most challenging atrial anatomies in RMN-guided AF ablation to navigate to include: the right inferior PV [8 (57%)] and the ridge and carina between left superior PV and LAA [2 (14%)].

### 5.7 Atrial fibrillation - ablation settings

While conventional settings during RF ablation involve applying low power for long times, a new setting applying high power in short duration, has recently been evaluated as safer and more effective (38–40), however long-term longitudinal outcomes are awaited. Overall, high-power short-duration lesions were significantly wider than and of similar depth compared to standard settings (38). These characteristics are most beneficial in PVI given the larger lesion diameter due to increased lesion-to-lesion uniformity and linear continuity.

In general, it is recommended by respondents to perform RMN-guided AF ablation in irrigated mode (LoC 14/14 (100%). The proposed ablation settings (including power, irrigation flow rate and application duration) of the respondents are presented in Table 5 and are described for various anatomic locations of the LA. There is no consensus amongst the RMN AF expert panel yet on the application of high power short duration applications. Whether high power settings, result in improved long-term AF recurrence rates, should be the focus of future research. The Ablation History feature may be used to guide lesion delivery, although the optimum values of applied energy in RMN have not yet been studied.

**Table 5:** Atrial fibrillation ablation settings.

Power		<30 W	30–44 W	≥45 W
LA posterior wall		1 (7%)	12 (86%)	1 (7%)
LA anterior wall		0 (0%)	11 (79%)	3 (21%)
LA carina		1 (7%)	10 (71%)	3 (21%)
LA ridges^a^		0 (0%)	8 (57%)	6 (43%)
Flow rate	Non-Irrigated	<10 ml/min	10–19 ml/min	≥20 ml/min
LA posterior wall	0 (0%)	0 (0%)	10 (71%)	4 (29%)
LA anterior wall	0 (0%)	0 (0%)	6 (43%)	8 (57%)
LA carina	0 (0%)	0 (0%)	8 (57%)	6 (43%)
LA ridges^a^	0 (0%)	0 (0%)	6 (43%)	8 (57%)
Duration	Continuous Dragging	<30 s	30–44 s	≥45 s
LA posterior wall	4 (29%)	6 (43%)	3 (21%)	1 (7%)
LA anterior wall	4 (29%)	5 (36%)	4 (29%)	1 (7%)
LA carina	4 (29%)	4 (29%)	5 (36%)	1 (7%)
LA ridges^a^	3 (21%)	6 (43%)	4 (29%)	1 (7%)

LA, left atrium.

^a^ Ridges include: RSPV 12 o’ clock, LSPV/LAA ridge, mitral anulus.

## 6 Recommendations on RMN-guided ventricular arrhythmia ablation

### 6.1 General

Ventricular arrhythmias (VA) are a major contributor to morbidity and mortality and present in a variety of forms, from single PVCs to sustained VT and VF (41). CA is an well-established treatment option for drug-refractory VA (41). The 2019 HRS/EHRA/APHRS/LAHRS expert consensus statement on catheter ablation of ventricular arrhythmias presents general recommendations on ablative treatment of VA (10). In general, VA ablation is a more complex procedure associated with prolonged radiation exposure and procedure times. Theoretically, RMN offers several advantages which are of particular benefit in VA ablation, including greater catheter stability, improved maneuverability, increased precision of RF energy delivery, decreased radiation exposure and improved outcomes. Although there is lack of large randomized trials comparing RMN with manual techniques, evidence from several observational and retrospective studies favor the advantages described above. Two meta-analysis evaluated the majority of the observational studies and concluded that RMN is safe and feasible in treatment of VA both in structural heart disease as in an idiopathic origin (6, 22). RMN-guided CA was superior to manual ablation [with or without contact force (CF) sensing catheters] in terms of acute success, adverse events and fluoroscopy exposure (6, 22). The first meta-analysis concluded that long-term VT recurrence rates were significantly lower patients treated with RMN (22). Whereas the other concluded that long-term VT recurrence rates were comparable between the two techniques, as well as in structural and non-structural heart disease subgroups (6). Further prospective (randomized) studies are expected to compare the efficacy between RMN and manual techniques (42).

RMN-guided VA ablation recommendations and general VA ablation considerations are presented in Tables 6, 7 respectively. Fifteen respondents consider the RMN system appropriate for every VA ablation [LoC 15/17 (88%)]. The RMN system is a well-suited technique to ablate ischemic VT [LoC 16/17 (94%)] and it is also appropriate for idiopathic VA [LoC 16/17 (94%)]. The RMN system is appropriate for VA originating from the outflow tracts (either RV or LV) [16/17 (94%)]. All respondents [LoC 17/17 (100%)] use the RMN system for all types of PVC ablation (whether originating from the right ventricle (RV), left ventricle (LV) or cusps). In addition, 16 respondents think the RMN system is suitable for epicardial ablation [LoC 16/17 (94%)].

**Table 6:** RMN specific recommendations on ventricular arrhythmia ablation.

Recommendation	Level of consensus	Symbol	References
General			
The RMN system is appropriate for all types of VT ablation	15/17 (88%)	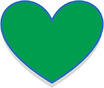	10, 50
The RMN system is suitable for ischemic VT ablation	16/17 (94%)	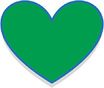	6, 22
The RMN system is suitable for idiopathic VT ablation	16/17 (94%)	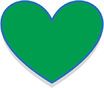	6, 22
The RMN system is suitable for outflow tract VA ablation	16/17 (94%)	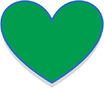	6, 22
The RMN system is appropriate for all types of PVC ablation, including those originating from the RV, LV and aortic cusps	17/17 (100%)	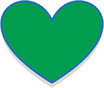	6, 22
The RMN system is appropriate for epicardial VA ablation	16/17 (94%)	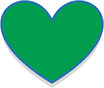	62–65
It is considered safe to use the RMN system in patients who have a pacemaker or ICD implanted	17/17 (100%)	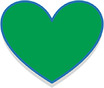	52, 53
The RMN system can be safely used in patients with subcutaneous ICD	14/17 (82%)	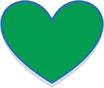	52, 53
Performing RMN ablations in patients on mechanical support with an implanted LVAD may be performed after careful consideration of other treatment options	10/17 (59%)	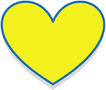	54, 55
RMN ablations in patients on mechanical support with ECMO are performed by a minority of respondents and should only be performed after careful consideration of treatment options	7/17 (41%)	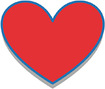	54, 55
Approach			
It is recommended that vascular access is attained by the operating electrophysiologist or fellow only.	17/17 (100%)	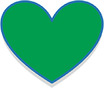	10
Both the transseptal and the retrograde aortic routes are considered suitable to approach left-sided targets with RMN	17/17 (100%)	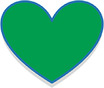	EO
Mapping			
The CARTO 3D mapping system is the first-choice mapping system for RMN-guided VA ablation	17/17 (100%)	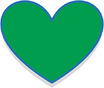	EO
Automation features			
Various automation features are used by a minority in RMN-guided VA ablation. These include: the Automap, Bullseye, Vector lock, Naviline and Click and Go features	2/17 (12%), 5/17 (29%), 2/17 (12%), 2/17 (12%), 5/17 (29%) resp.	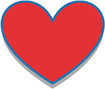	EO
The Vdrive is used by a minority for RMN-guided VA ablation	4/17 (24%)	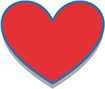	EO
RMN settings			
It may be considered to use a magnetic field strength of 0.1 T	13/17 (76%)	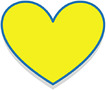	EO
It is rarely needed to tilt the magnets for larger patients or steeper imaging angles	3/17 (18%)	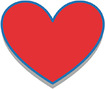	EO
It is recommended to use e-Contact Module during RMN-guided VA ablation, to evaluate whether the ablation catheter is in (optimal) contact with the myocardial wall tissue or not	9/10 (90%)	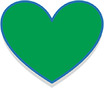	32, 33
It is recommended to use the Ablation History feature during RMN-guided VA ablation, which is used real-time to evaluate the completeness of applications	14/17 (82%)	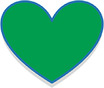	EO
Sheaths, catheters and manipulation			
In case of a retrograde transaortic LV approach, it may be considered to use a short femoral sheath	11/17 (65%)	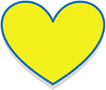	10, 11
In case of a retrograde transaortic LV approach, a minority use a long sheath past the aortic valve	2/17 (12%)	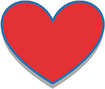	10, 11
In case of a transseptal LV approach, the use of one of the following sheaths may be considered: SL0, SL1 or steerable sheath	12/17 (71%)	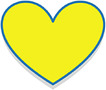	10, 11
It may be considered not to cross the septum without sheath support	11/17 (65%)	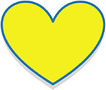	EO
A minority use the introducer (i.e., haemo-adapter) to insert the RMN catheter into the sheath	5/17 (29%)	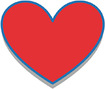	EO
Sheath manipulation is used by a minority during a RMN-guided VA ablation	8/17 (47%)	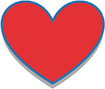	EO
The SmartAblate Stockert ablation generators are recommended for RMN-guided VA ablation	17/17 (100%)	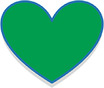	11
The Navistar Thermocool RMT catheter is the most frequently catheter used in RMN-guided VA ablation, but other RMT catheters can be used with the system as well.	15/17 (88%)	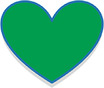	EO
It may be considered to navigate towards anatomical areas that are difficult to reach, prior to starting the ablation	12/17 (71%)	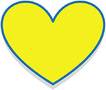	EO
Advancing and retraction of the RMN catheter is recommended by rolling the mouse wheel, although a minority uses the key pad or joy stick	15/17 (88%)	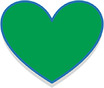	EO
Epicardial ablation			
The RMN system is appropriate to use in the epicardial space	16/17 (94%)	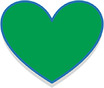	62–65

ECMO, extracorporeal membrane oxygenation; ICD, implantable cardioverter defibrillator; LV, left ventricle; LVAD, left ventricular assist device; PVC, premature ventricular contractions; RMN, robotic magnetic navigation; RV, right ventricle; VA, ventricular arrhythmia; VT, ventricular tachycardia.

**Table 7:** General considerations on ventricular arrhythmia ablation.

Recommendation	Level of consensus	Symbol	References
General			
The majority of respondents perform VA ablation under conscious sedation or general anesthesia, but its use should be weighted upon individual needs, the inducibility of VA and the target of ablation	13/17 (76%)	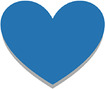	10
Approach			
TSP is most frequently performed either at the central part of the foramen ovale, or at a more low anterior position	12/17 (71%)	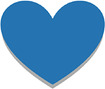	EO
TSP is commonly performed with TOE or ICE guidance	17/17 (100%)	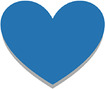	9, 26, 28
The majority of respondents use ICE in preference to TOE to guide TSP	10/17 (59%)	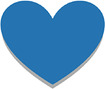	EO
Mapping			
All respondents perform high-density electro-anatomic mapping to identify VA substrate	17/17 (100%)	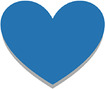	10, 60, 61
Respiratory compensation is used by the majority during mapping	14/17 (82%)	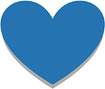	EO
The CARTO Sound Map is used by the majority of respondents and is considered useful to identify papillary muscles, outflow tract and aortic cusps.	10/17 (59%)	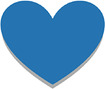	EO
VA ablation strategy			
Homogenization of the scar is the most frequently used treatment strategy for ischemic VT ablation	13/17 (76%)	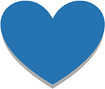	10
Epicardial ablation			
The majority of respondents achieve pericardial access by an anterior approach	13/17 (76%)	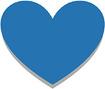	66
The Sosa technique is used by the majority of respondents to gain pericardial access	11/17 (65%)	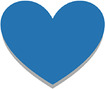	66

FAM, fast anatomical mapping; ICE, intracardiac echocardiography; PVC, premature ventricular contractions; RMN, robotic magnetic navigation; TOE, transesophageal echocardiography; TSP, transseptal puncture; VA, ventricular arrhythmia; VT, ventricular tachycardia.

It is considered safe to use the RMN system in patients who have a pacemaker or ICD implanted (43, 44). The RMN VA expert panel also considers the RMN safe in patients with pacemaker or ICD [LoC 17/17 (100%)] and in patients having a subcutaneous ICD [LoC 14/17 (82%)]. One prospective study reported no relevant changes in lead parameters or device programming after the RMN procedure (43). Almost half of the devices included in this particular study switched to asynchronous stimulation during the procedure, without clinical adverse events (43). The expert panel therefore advises that MR conditional cardiac devices are programmed in MRI mode. In implanted devices without specific MR mode, the device should be programmed in the mode that gives minimal interference (43). The pacing mode should be carefully and individually selected, especially in patients who are completely pacing dependent. In these patients, a conceivable approach is the placement of a temporary pacing lead and subsequently programming the implanted device to “no capture” by reducing the output to the lowest programmable setting, before the magnets are activated.

There is limited experience on the use of the RMN system in patients on mechanical circulatory support, such as left ventricular assist device (LVAD) (including LVADs employing magnetically levitated pump) (45, 46). Performing RMN ablations in patients on mechanical support may be performed after careful consideration of other treatment options. The VA expert panel members reported certain positive experience of RMN-guided ablation in patients with a LVAD [performed by 10 (59%) respondents] and extracorporeal membrane oxygenation (ECMO) [performed by 7 (41%) respondents]. Further studies are needed to establish clear protocols for these special populations to ensure safety and efficacy.

The expert consensus statement on catheter ablation of VA provides recommendations on the use of conscious sedation and general anesthesia during CA of VA, which also apply to RMN (10). It is recommended to provide sedation of variable depth and analgesia, or general anesthesia during mapping and ablation of VA. Moreover, it is considered reasonable to avoid general anesthesia and deep sedation in patients with idiopathic VA, particularly if the arrhythmia is thought to be catecholamine-sensitive or hasn't been inducible during a previous procedure (10).

Regarding RMN-guided VA ablation, most respondents (13 (76%) perform the majority of their procedures either with general anesthesia or conscious sedation depending upon clinical requirements, inducibility of VA and the ablation targets. No sedation, conscious sedation or general anesthesia is employed for the majority of VA ablation procedures by four (24%), ten (59%) and three (36%) respondents, respectively. Only few respondents [5 (29%)] have an anesthesiologist present during all VA ablation procedures, as for instance many procedures are performed without sedation or sedation administered by a trained nurse.

### 6.2 Approach

General recommendations on the vascular access and approach in VA ablation are described extensively elsewhere (10).

LV access can be achieved through either an antegrade transseptal or a retrograde transaortic approach, depending on patient specifics and operator preference (10). Transseptal access for instance can be preferential in patients with mechanical aortic valve or severe peripheral vascular disease (10). In general, most areas of the endocardial LV can be accessed by either approach, but in manual procedures, the degree of contact force can vary significant depending on the approach chosen (47). Manual ablation catheters are confined to uni- or bidirectional movement using pull wires, and the stability of catheter-tissue contact and contact force, is therefore subject to entry angle and site of the chamber of interest (48). In contrast, magnetic navigation catheters by design are more flexible and pulled towards the area of interest by magnetic force. This ensures enhanced maneuverability that makes reach of difficult anatomical structures possible (1, 49). Consequently, RMN-guided CA of left-sided targets is less susceptible to the chosen approach (50). Therefore, both the transseptal and the retrograde aortic route are considered suitable to approach left-sided targets by the RMN expert panel. Two respondents (12%) use a retrograde transaortic approach for >50% of their VT ablation procedures. Others only use this in a minority of VT ablation procedures [25%–50% retrograde approach: 2 (12%), 10%–25% retrograde approach: 3 (18%), <10% retrograde approach: 10 (59%)]. The majority of respondents [12 (71%)] perform TSP at a central septal or at a more inferior anterior septal location. All respondents [17 (100%)] perform TSP with TOE or ICE guidance. The majority of respondents [10 (59%)] use ICE to guide TSP in preference to TOE.

### 6.3 Mapping

As mentioned previously, the RMN system has full mapping integration with the CARTO 3D mapping system (Biosense Webster Inc.) and the AcQMap (Acutus Medical Inc.) mapping systems. As the AcQMap system is confined to atrial procedures, the CARTO 3D mapping system (Biosense Webster Inc.) is considered the first-choice fully integrated mapping system for RMN-guided VA ablation [LoC 17/17 (100%)] (Figure 2). The EnSite NavX (Abbott) and the Rhythmia HDx (Boston Scientific, Marlborough, MA, USA) mapping systems are occasionally used as well, but only in parallel (not fully integrated) fashion.

During mapping, a high median FAM resolution is used by the majority of respondents (median 18.5 (IQR 13.8–20.0, range 10.0–20.0). In addition, the majority of the RMN expert panel [14 (82%)] use respiratory compensation during mapping. The CARTO sound map setting is used by ten respondents (59%). Respondents specified that it can be useful to identify papillary muscles, outflow tract and aortic cusps.

### 6.4 Automation features and Vdrive

Various automation features have been developed for RMN, however they are used by a minority of respondents in standard RMN-guided VA ablation. These include: the Automap feature [LoC 2/17 (12%)], the Bullseye feature [LoC 5/17 (29%)], the Vector lock feature [LoC 2/17 (12%)], the Naviline feature [LoC 2/17 (12%)] and the Click and Go feature [LoC 5/17 (29%)].

In general, the Vdrive is not used as standard for care during RMN-guided VA ablation because of various reasons described previously (see RMN-guided AF recommendations). Vdrive is sporadically used by 4 respondents (24%). Four (24%) never use it but have the feature installed, whereas 9 respondents (53%) do not have it installed.

### 6.5 RMN settings

In most cases it may be considered to preferentially use a magnetic field strength of 0.1 T [LoC 13/17 (76%)] instead of 0.08 T. However, the setting of 0.08 T is applicable to any patient (especially patients with increased axial chest dimensions and/or obesity because of more distance between the magnets). It is rarely needed to tilt the magnets for larger patients or steeper imaging angles [LoC 3/17 (18%)]. A median CAS step size of 2.5 (IQR 1.8–3.3, range 1.0–4.0) mm is recommended.

The E-Contact Module and Ablation History features were described previously (see RMN-guided AF recommendations). It is recommended to use e-Contact Module during RMN-guided VA ablation [LoC 9/10 (90%)], to evaluate whether the ablation catheter is in (optimal) contact with the myocardial wall tissue or not. Seven respondents (41%) do not have this feature installed yet because it not being FDA approved for use in the U.S.A. at the time of writing. It is recommended to use the Ablation History feature during RMN-guided VA ablation (LoC 14/17 (82%), which is used in real-time to evaluate the applied therapy as well as the completeness of ablation.

### 6.6 Sheaths, catheters and manipulation

The indication of various deflectable sheaths is described in-detail elsewhere (10, 11). Regarding RMN-guided procedures, it may be considered to use a short femoral sheath in case of retrograde transaortic approach [LoC 11 (65%)], although some respondents [8 (47%)] comment that they sometimes also use a long sheath up onto the aorta. A minority use a long sheath past the aortic valve [LoC 2/17 (12%)]. The following types of sheaths are used to guide the RMN catheter in a retrograde transaortic approach: short femoral sheath [11 (65%)], SL0 sheath (Abbott) [3 (18%)], SR0 (Abbott) [1 (5.88%)], LAMP sheath (Abbott) [1 (6%)]. In case of a transseptal LV approach, the use of one of the following sheaths may be considered: SL0, SL1 or steerable sheath [LoC 12/17 (71%)]. The following sheaths are used by others: Mullins sheath (Cook Medical, Bloomington, IN, USA) (2 (12%) and the MobiCath sheath (Biosense Webster) [2 (12%)]. It is advised not to cross the septum without sheath support by eleven respondents [LoC 11/17 (65%)].

A minority use the introducer (i.e., haemo-adapter) to insert the RMN catheter into the sheath [LoC 5/17 (29%)] in RMN guided VA ablation. There is controversy whether the sheath has to be manipulated during a RMN-guided VA ablation procedure to support catheter movement: eight respondents do so (47%), but others rarely to never manipulate their sheath [LoC 9/17 (53%)].

General recommendations on the ablation generators used in RF CA are presented elsewhere (11). It is recommended to use one of the various generations of the SmartAblate Stockert ablation generators (Johnson&Johnson/Biosense Webster) [LoC 17/17 (100%)]. The Navistar Thermocool RMT catheter (Biosense Webster) is recommended for RMN-guided VA ablation [LoC 15/17 (88%)], others [3 (18%)] also use the Celsius Thermocool RMT catheter (Biosense Webster).

It may be considered to navigate towards anatomical areas that are difficult to reach prior to starting the ablation [LoC 12/17 (71%)]. Advancement and retraction of the RMN catheter is recommended by rolling the mouse wheel [LoC 15/17 (88%)], but can also be done by using the keypad [LoC 3/17 (18%)] or joy stick [LoC 6/17 (35%)]. It may be considered to “loop” or “pin and roll” the catheter to navigate under the valve [LoC 11/17 (65%)].

Respondents propose the following solutions for anatomical regions that are difficult to navigate to: looping of the catheter [5 (29%)], a combination of applying different vectors and push/pull the catheter [6 (35%)] and sheath manipulations [4 (24%)]. Respondents mention that the most challenging anatomies to navigate to include: the regions just under the valves and the papillary muscles.

### 6.7 VA ablation strategy

The 2019 HRS/EHRA/APHRS/LAHRS expert consensus statement on catheter ablation of ventricular arrhythmias provides recommendations on the mapping and ablation strategy for the wide spectrum of VA (10). The recommended strategies in general also apply to RMN-guided VA ablation. The RMN VA expert panel performs scar homogenization most frequently as treatment strategy in ischemic VT [13 (76%)]. For focal VA (either nonsustained or sustained VT or PVCs, typically with idiopathic origin) activation and/or pace mapping are considered adequate to guide ablation (10).

Noninducibility of VT by programmed electrical stimulation (PES) after ablation is a recognized endpoint of the VT ablation procedure and predictor for VT recurrence in patients with structural heart disease (10). For focal arrhythmias, a reasonable endpoint is termination of VT or the complete elimination of PVCs, together with subsequent noninducibility of VT or PVCs by PES and/or catecholamine infusion (when episodes were inducible by this prior to ablation) (10).

### 6.8 Epicardial ablation

The RMN system is considered feasible and safe to use in the epicardial space (51). RMN has a particular advantage in the epicardial space because it allows a more direct approach to map the epicardial surface by altering the magnetic vector (52). By directing the magnetic vector towards the surface of the heart during RF applications, damage to structures adjacent to the heart can be prevented (53). A multicenter study evaluating efficacy and safety of RMN-guided epicardial VA ablation (the EPINAV study, ClinicalTrials.gov Identifier: NCT04171479) is expected soon. Any pericardial CA procedure is associated with higher risk for procedural complications and therefor careful selection of patients is warranted (54).

In total, sixteen respondents (94%) use the RMN system for epicardial VA ablation. The RMN system is considered appropriate to use in the epicardial space [LoC 16/17 (94%)]. The experience of respondents with RMN-guided epicardial ablation is markedly dispersed: 10 (59%) performed <5 procedures in the past 12 months; 5 (29%) performed 6–15 procedures; 5 (29%) performed 15–30 procedures and 1 (6%) performed >30 procedures. Respondents reported the following indications for epicardial ablation: ischemic VT 8 (47%), PVC 3 (18%), Arrhythmogenic right ventricular dysplasia (ARVD) 14 (82%), non-ischemic dilated cardiomyopathy (DCM) 8 (47%), hypertrophic cardiomyopathy (HCM) 1 (6%), post-myocarditis 2 (12%), cardiac sarcoid 3 (18%) and Brugada syndrome 2 (12%).

Pericardial access is most often achieved with a subxiphoidal, pericardial puncture using a Tuohy needle or similar, guided by contrast injection and fluoroscopic imaging, to demonstrate the position of the needle tip (Sosa technique) (55). The majority of the RMN VA expert panel (11 (65%) also use the Sosa technique. The majority of respondents achieve pericardial access by an anterior approach [13 (76%)]. A posterior approach [8 (47%)] or surgical approach [1 (6%)] are used in a minority of cases. Bubble injection combined with ultrasound guidance is used by a minority [2 (12%)]. There is no consensus on the type of sheaths used for the pericardial space, though most operators [9 (53%)] use a short steerable sheath.

Imaging of the coronary arteries with coronary angiography or preprocedural CT angiography is advised to accurately localize the ablation catheters position with respect to the position of the coronary arteries (10). In addition, pre-procedural imaging can be of use to inspect the course of the phrenic nerve (10). In case of emergent visualization of coronary arteries during the ablation, it is possible to temporarily put the magnets in the (semi)stowed position to allow angulation of the fluoroscopy system. Regarding RMN-guided epicardial VA ablation, there is no consensus on the preferential visualization of coronary arteries, which either can be marked with point annotations on the electro-anatomic map [5 (29%)], or with other imaging integration techniques (including preprocedural CT scan and coronary angiography during the ablation procedure).

### 6.9 VA - ablation settings

Considerations on the ablation power, temperature limits and irrigation, as well as contact force, of manual RF CA have been well-described (10). An impedance drop >10 ohms or a contact force >10 g is commonly used as a target for RF energy delivery (10). RMN systems were reported to create a reasonable stable contact force level of 6.1 ± 1.4 g (i.e., when used without a long sheath) (36). Nevertheless, the lesion volume, width and depth of RMN-guided RF applications in an animal ventricular ablation model were not significantly different compared to manual applications applied with a variety of contact forces (ranging from 5 g–20 g) (56). This suggests that RMN might not rely on the level of force imposed but on the stable contact level and orientation of the catheter as determined by the magnetic field. Therefore, contact force measurements are considered not that informative in guiding RMN lesion delivery.

Regarding RMN, it is recommended to perform VA ablation in irrigated mode [LoC 17/17 (100%)]. The ablation settings (including power settings, irrigation flow rate and application duration) advised by respondents are presented in Table 8 and differ between various anatomical sites. The Ablation History feature [calculating the applied energy and duration (W*s) per location] may be used to guide lesion delivery. Until today there are no studies evaluating the optimum of the applied energy in RMN.

**Table 8:** Ventricular arrhythmia ablation settings.

Power		<30 W	30–44 W	≥45 W	Missing
Free wall		0 (0%)	7 (41%)	10 (59%)	0 (0%)
Outflow tract		0 (0%)	12 (71%)	5 (29%)	0 (0%)
Septum		0 (0%)	3 (18%)	14 (82%)	0 (0%)
Papillary muscle		0 (0%)	6 (3%)	11 (65%)	0 (0%)
Close to conduction system		5 (29%)	12 (71%)	0 (0%)	0 (0%)
Close to coronary artery		6 (35%)	7 (41%)	2 (12%)	2 (12%)
Epicardial		0 (0%)	9 (53%)	6 (35%)	2 (12%)
Flow rate	Non-irrigated	<10 ml/min	10–19 ml/min	≥20 ml/min	Missing
Free wall	0 (0%)	0 (0%)	3 (18%)	12 (71%)	2 (12%)
Outflow tract	0 (0%)	0 (0%)	4 (24%)	11 (65%)	2 (12%)
Septum	0 (0%)	0 (0%)	3 (18%)	12 (71%)	2 (12%)
Papillary muscle	0 (0%)	0 (0%)	3 (18%)	12 (71%)	2 (12%)
Close to conduction system	1 (6%)	1 (6%)	4 (24%)	9 (53%)	2 (12%)
Close to coronary artery	0 (0%)	0 (0%)	3 (18%)	10 (59%)	4 (24%)
Epicardial	0 (0%)	5 (29%)	3 (18%)	6 (35%)	3 (18%)
Duration	Continuous dragging	<30 s	30–59 s	≥60 s	Missing
Free wall	1 (6%)	1 (6%)	4 (24%)	8 (47%)	3 (18%)
Outflow tract	0 (0%)	0 (0%)	6 (35%)	10 (59%)	1 (6%)
Septum	0 (0%)	1 (6%)	2 (12%)	11 (65%)	3 (18%)
Papillary muscle	0 (0%)	0 (0%)	2 (12%)	8 (47%)	7 (41%)
Close to conduction system	0 (0%)	1 (6%)	7 (41%)	7 (41%)	2 (12%)
Close to coronary artery	0 (0%)	2 (12%)	5 (29%)	6 (35%)	4 (24%)
Epicardial	1 (6%)	1 (6%)	5 (29%)	6 (35%)	5 (29%)

## 7 Conclusions

This is the first position paper to evaluate and present best practices for RMN-guided CA. The statement summarizes the currently used strategies and provides recommendations on treatment of AF and VA with RMN-guided CA and addresses best practices in set-up of the patient, compatibility of the RMN system with other systems, procedural set-up, materials and the approach, target and endpoint of ablation. The recommendations offer a unique resource of advice for physician who are starting to use RMN in the treatment of cardiac arrhythmias. However, the recommendations should be used as framework, and other approaches may be used if the operator has found them helpful (e.g., for RMN catheter manipulation).

There are still significant gaps in the clinical evidence that need to be addressed in future research. Many recommendations are based on expert consensus rather than high-quality evidence. While expert opinions are valuable, they can introduce subjective biases, particularly in the absence of rigorous comparative data. Specifically, high volume randomized trials are warranted comparing procedural characteristics and outcomes between RMN and other ablation techniques and should focus on long-term results in particular. In addition, future studies on the combined use of RMN with novel energy forms, such as high-power-short-duration radiofrequency energy and pulsed-field ablation, and cost-effectiveness are awaited.

In addition, there are still challenges to overcome with RMN in catheter design, mapping and advanced automation. The development of novel catheters compatible with RMN is stimulated, however at present there are limited types available. It would be of value to expand the integration of RMN with other imaging techniques, mapping systems and high-density multipolar mapping. These matters are crucial for a more widespread adoption of the technology. These implementations will contribute to the future development of fully automated algorithms for catheter navigation and ablation.

In conclusion, the results of this survey-based analysis of expert opinion provide unique guidance on the best-practices of RMN-guided treatment of AF and VA.
